# Circulating Programmed Death-1 as a Marker for Sustained High Hepatitis B Viral Load and Risk of Hepatocellular Carcinoma

**DOI:** 10.1371/journal.pone.0095870

**Published:** 2014-11-26

**Authors:** Hsiang-Yun Cheng, Pei-Jen Kang, Ya-Hui Chuang, Ya-Hui Wang, Meng-Chin Jan, Chih-Feng Wu, Chih-Lin Lin, Chun-Jen Liu, Yun-Fan Liaw, Shi-Ming Lin, Pei-Jer Chen, Shou-Dong Lee, Ming-Whei Yu

**Affiliations:** 1 Institute of Epidemiology and Preventive Medicine, College of Public Health, National Taiwan University, Taipei, Taiwan; 2 Department of Clinical Laboratory Sciences and Medical Biotechnology, College of Medicine, National Taiwan University, Taipei, Taiwan; 3 Department of Gastroenterology, Ren-Ai Branch, Taipei City Hospital, Taipei, Taiwan; 4 Division of Gastroenterology, Department of Internal Medicine, National Taiwan University Hospital and National Taiwan University College of Medicine, Taipei, Taiwan; 5 Liver Research Unit, Chang Gung Memorial Hospital, Chang Gung University College of Medicine, Taipei, Taiwan; 6 Division of Gastroenterology, Department of Medicine, Taipei Veterans General Hospital, Taipei, Taiwan; 7 Cheng Hsin General Hospital, Taipei, Taiwan; Yonsei University College of Medicine, Republic of Korea

## Abstract

**Objective:**

Recent evidence indicates a crucial role of the immunoinhibitory receptor programmed death-1 (PD-1) in enforcing T-cell dysfunction during chronic viral infection and cancer. We assessed the impact of circulating soluble PD-1 (sPD-1) levels on long-term dynamics of hepatitis B virus (HBV) load and hepatocellular carcinoma (HCC) risk.

**Methods:**

In a case-cohort study on longitudinal analysis of viral load within a cohort of 2903 men chronically infected with HBV, followed up from baseline (1989–1992) through 2010, we determined sPD-1 levels in baseline plasma with enzyme-linked immunosorbent assay from 126 men who subsequently developed HCC and 1155 men who did not develop HCC. To evaluate whether patients' characteristics involved the use of sPD-1 as a biomarker, sPD-1 was also tested in 614 newly-diagnosed patients with HBV-related HCC recruited from a multicenter study for comparison with the 1155 noncases in the case-cohort study.

**Results:**

Plasma quartile levels of sPD-1 were positively associated with HCC risk for men in the case-cohort analysis (vs. quartile 1: adjusted odds ratios [95% confidence intervals] for quartile 2-quartile 4 were 1.51 [0.75–3.03], 2.15 [1.12–4.13], and 2.29 [1.20–4.38], respectively), and in the case-control study regardless of age-of-onset and clinical stage. Furthermore, we found longitudinal effect of elevated sPD-1 levels to maintain higher viral load for 4 or more years, with greater and more prolonged effect among HBV genotype C- vs. non-C-infected participants. High levels of viral load and sPD-1 (vs. absence of both) was associated with a 6.29-fold increase in risk of HCC, and combining both conditions with HBV genotype C yielded an odds ratio of 30.47 with significant additive interaction (relative excess risk due to interaction: 27.08 [95% confidence interval = 8.76–45.41]).

**Conclusions:**

Our data suggest plasma sPD-1 as an important immune-related marker for assessment of HBV activity and HCC risk.

## Introduction

Chronic hepatitis B virus (HBV) infection is a major cause of hepatocellular carcinoma (HCC) worldwide [1]. HBV replication determined by viral load is key to liver injury and disease progression [2]–[4]. The viral load and the rate of disease progression among persons chronically infected with HBV vary widely, depending on the interaction between virus and the host's immune system [5]–[7]. In contrast to the abundant data on viral factors; however, the value of measurements of immunological markers for hepatitis B has been rarely studied [7].

In chronic viral infection, the persistent exposure to high concentrations of viral antigens leads to various degrees of T-cell functional impairments referred to as T-cell exhaustion [8], [9]. Recent animal models of chronic viral infection indicate that the interaction between programmed death-1 (PD-1), a negative regulator of activated T cells, and its ligands (PD-L) plays a critical role in T-cell exhaustion [10], [11]. In patients with chronic hepatitis B, PD-1 expression on CD8+ T cells correlates with viral load, and reduction in viral load by antiviral therapy is accompanied by decrease in PD-1 expression [12]. Further studies indicate that *in vitro* blocking PD-1-PD-L interaction results in functional restoration of HBV-specific CD8+ T cells [9]. High levels of PD-1 expression is also found on tumor-infiltrating CD8+ T cells in multiple solid tumors, including HCC, and there is evidence for a role of the PD-1-PD-L1 pathway involved in the escape from host immune system in cancer [13]–[16].

In addition to the membrane-bound PD-1 on T cells, there is circulating, soluble PD-1 (sPD-1) [17]. Little is known about the origin and physiological functions of sPD-1, but it has been already used as an antagonistic of PD-1 signaling in experimental studies [17], [18]. Several lines of evidence implicate a role of soluble receptors in regulating inflammatory and immune events by functioning as agonists or antagonistics of cytokine signaling [19], [20]. Therefore, an association between sPD-1 and HBV-related HCC development can be assumed.

In this report, we investigated whether baseline plasma sPD-1 levels impact on long-term HBV viral load and subsequent risk of HCC in hepatitis B surface antigen (HBsAg)-positive individuals who had HBV monoinfection or HBV/hepatitis C virus (HCV) dual infection. As liver cirrhosis is considered preneoplastic condition, we also included liver cirrhosis as an endpoint in analyses. By using an independent case series with wider range of clinical factors, we further assessed whether sPD-1 was detectable at a higher level in plasma from patients with existing HBV-related HCC in comparison with noncases, and whether patients' characteristics in terms of gender, age-of-onset, and clinical variables involved the use of sPD-1 as a biomarker.

## Materials

**Figure 1:**
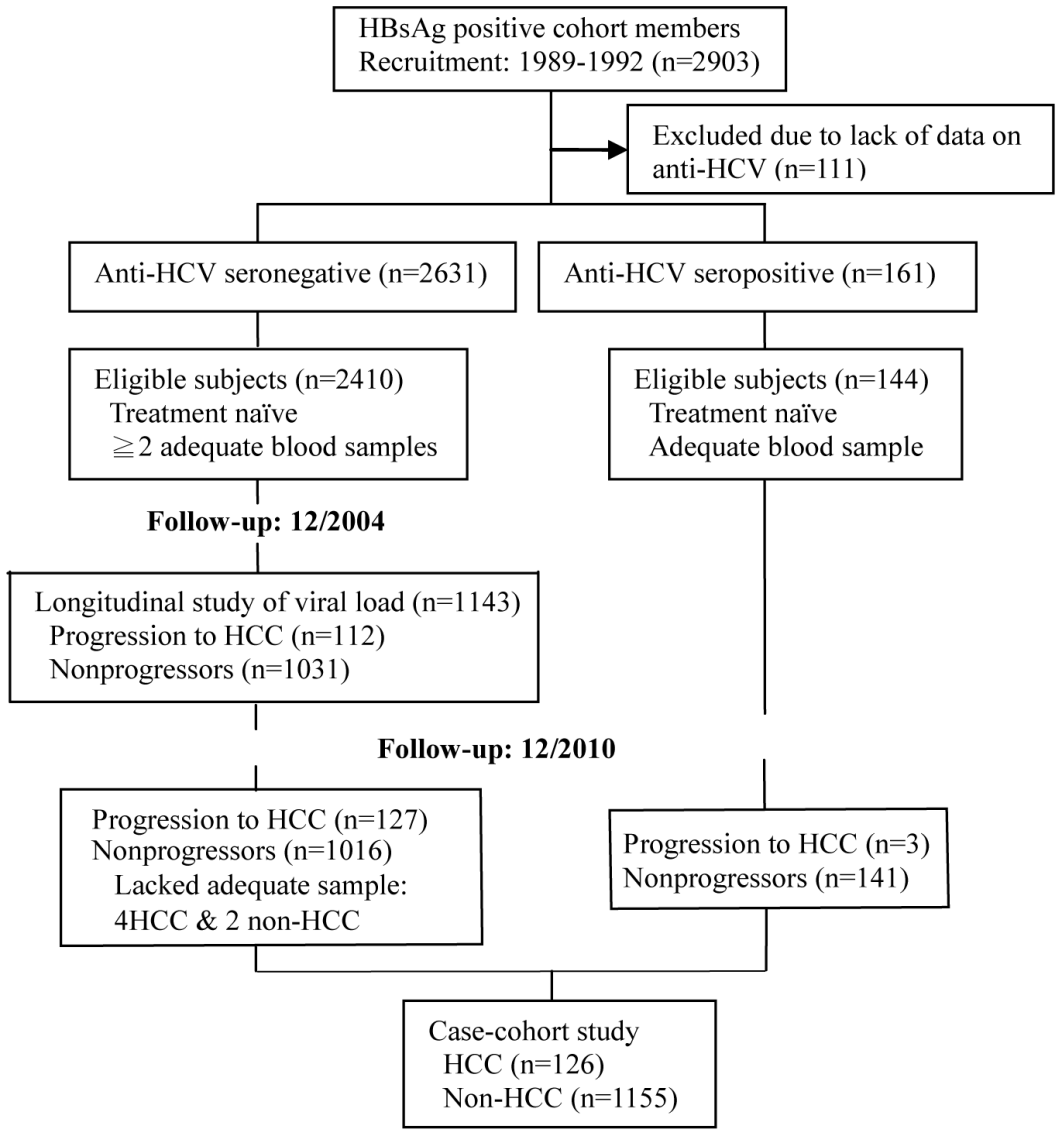
Flow of participant recruitment and follow-up in the case-cohort study.

This study was conducted with 1281 study subjects recruited from a case-cohort study (Figure 1) and an independent case series of 614 patients with existing HCC recruited from a multicenter study. It was approved by the research ethics committee at the College of Public Health, National Taiwan University and all participants provided written informed consent.

### Design and population of case-cohort study

The cohort included 2903 HBsAg-positive men aged 30–65 years who were free of HCC at enrollment during routine free physical examination in 1989–1992 at Government Employee Central Clinics [21]. All participants had a baseline clinic visit at which they underwent a physical examination consisting of HBsAg, alanine aminotransferase [ALT], and α-fetoprotein, and provided a blood specimen and risk factor information (including lifestyle and medical history) according to a structured questionnaire by trained research assistants. Participants were instructed to schedule blood sample collection in the morning following an overnight fast, and standardized procedure was used for the collection and storage of blood samples. After recruitment, participants were invited to return for follow-up examinations (including ultrasound and liver biochemical tests) every year and more frequently if indicated. Liver ultrasound was performed routinely since 1993 for the detection of liver abnormalities, including fatty liver, cirrhosis, and HCC.

Participants returned for follow-up on a voluntary basis. Those who had abnormal liver biochemical tests or ultrasonographic features during follow-up were informed in a report of results and advised to receive further clinical evaluation. At the end of all the follow-up examinations, 81.2% of participants who subsequently developed HCC and 81.3% of those who did not had at least one return clinical visit for ultrasonography measurement. The median number of total visits is 4 (interquartile range:2–7) and 7 (interquartile range:4–9) for the two groups, respectively. Because ultrasonography screening is known to have a sensitivity of approximately 60% for early HCC and HCC detection strongly depends upon patients' compliance with follow-up and referrals for confirmation testing, cancer occurrence and vital status data were also obtained by linking data with the computer files of national death certification and cancer registry systems to complement follow-up examination for achieving complete ascertainment of cases.

In Taiwan, reimbursement under the National Health Insurance program for antiviral therapy for hepatitis B patients meeting certain criteria began on October 2003. Since 2002, we expanded our follow-up interview questionnaire to include questions about any antiviral therapy the participant might have received. Because the criteria for reimbursement are strict, till the end of the follow-up (i.e., 2010) the proportion of study subjects who returned for examinations and had a history of antiviral therapy was only ∼2%, which is similar to that recently reported by studies using the Taiwan National Health Insurance Research Database.

The present study used a case-cohort design and included 126 HCC cases and 1155 noncase subjects. The sample consisted of all HBV/HCV dual-infected men who had adequate baseline samples (n = 144) in the full cohort, and approximate 47% of the eligible HBV-monoinfected men who were selected using the case-cohort method of sampling. The enrollment of the HBV-monoinfected subjects has been reported elsewhere [3]. Briefly, in 2004, we chose a random subcohort of 1084 men, including 53 cases of HCC identified by then, from the full cohort. Cohort members were eligible for inclusion if they had ≥2 blood samples after study entry and no prior history of antiviral therapy. Fifty-nine cases of HCC outside the subcohort were added to the sample. Four HCCs and two noncases lacked adequate samples for the sPD-1 testing, leaving 1137 HBV-monoinfected men.

In this report, incident HCC cases were identified based on follow-up examination or linking participants to the national cancer registry databases through 2006, while vital status and cause-of-death code were obtained through linkage with death registry databases available for years 1989–2010. Among the 126 HCC cases in the analysis, 4 were only identified through death certificates. The 122 of the 126 HCC cases were ascertained by the following criteria: histopathologic findings, positive lesion detected by at least 2 imaging techniques (ultrasound, angiography, or computed tomography), or a single imaging technique plus a serum α-fetoprotein level of ≥400 ng/ml.

### Analysis of an independent case series

We also conducted a case-control analysis, comprising 614 hospital-based cases and 1155 noncases from the case-cohort study as controls. This part of study aimed to recruit a wider range of patient subgroups each with sufficient sample size for stratified analysis, which allowed evaluation of whether the results might be applicable to different subgroups of patients with HCC. Newly-diagnosed patients with HCC meeting the following criteria were recruited from a multicenter study [22]: 1) age of 20 to 75 years; 2) having positive HBsAg; and 3) no prior history of other cancers. From 1290 eligible patients enrolled in 1997–2004, we generated a random sample by selecting approximately equal number of early- (i.e., solitary tumor ≤5 cm or <4 lesions and none >3 cm) and late-stage HCC patients [22]. Finally, 295 early- and 319 late-stage patients were chosen to be analyzed for sPD-1.

### Laboratory assays

HBV genotype was determined by multiplex polymerase chain reaction, and plasma HBV-DNA levels were measured by TaqMan assay with a detection limit of 215 copies/mL as described previously [2], [3]. For the longitudinal analysis of viral load, we analyzed all frozen plasma samples taken during a period of up to 16 years before diagnosis. Seventy-one percent (908/1281) of the participants underwent 6 or more repeated testing (interquartile range = 5–9). A total of 7672 samples from HBV-monoinfected men and 984 from HBV/HCV dual-infected men were tested for viral load. sPD-1 levels were assayed using baseline plasma with R&D Systems enzyme-linked immunosorbent assay kit (Minneapolis, MN). The intra-assay and inter-assay coefficients of variance for sPD-1 were less than 10%.

### Statistical analysis

Comparisons between groups were performed using Wilcoxon rank sum test for continuous data, and Fisher's exact test for categorical data. HBV-DNA data were log_10_-transformed to normalize the distributions before analyses. We assigned a value of 215 copies/mL to samples that had undetectable HBV DNA. Separate logistic regression models were built to test whether plasma sPD-1 is associated with the occurrence of HCC or liver cirrhosis, and multivariable odds ratios (ORs) and their 95% confidence intervals (CIs) were estimated. Trends in ORs across sPD-1 quartiles (Q) were tested in logistic regression as an ordinal variable coded as 1 to 4, respectively, to Q1–Q4. All models were adjusted for age, smoking, and alcohol consumption. Cigarette smoking was defined as smoking at least 4 days a week for more then one year. Alcohol consumption was defined as consumption of any alcoholic beverage at least once a week for more than one year. Family history, BMI, ALT, and HBV-related factors are important HCC risk factors [2]–[5], [21] and potential confounders, yet detailed assessment of all these variables was available only for the case-cohort study. Uncontrolled confounding give rise to biased effect estimates. To assess the magnitudes of these biases, models were repeated with additional adjustment for other factors where appropriate, and the ORs for sPD-1 were compared. We illustrate the results from this sensitivity analysis for potential confounding effects to establish the robustness of our results. To estimate the degree of interactions between risk factors, we calculated relative excess risk due to interaction (RERI); attributable proportion due to interaction (AP); and synergy index [23]. Given the similar effect sizes of the associations between Q3 and Q4 and the risk of HCC, in analysis of interactions with sPD-1, Q3 and Q4 were combined and labeled “high sPD-1”, whereas Q1 was combined with Q2 and labeled “low sPD-1”.

In analysis of the effect of baseline sPD-1 levels on serial viral load, we evaluated viral load as continuous and as binary (defined by a predetermined cutoff of 4.39 log copies/mL [2], [3]) outcome variables. Smoothed scatterplots for the relationship between viral load and time were generated using locally weighted regression (LOESS). We used linear mixed model adjusted for age to determine the effect of high sPD-1 (>282 pg/mL) on the change in viral load treated as a continuous variable over time. To assess the effect of high sPD-1 on the tendency to maintain a high level of viral load over time, we fitted generalized mixed models for binary longitudinal outcomes using SAS Proc GLIMMIX, with inclusion of age and the time at follow-up assay as covariates. Because HBV genotype affects viral load [2], [3], all such analyses were performed according to HBV genotype. Statistical computing was conducted using SAS version 9.2 (SAS Institute, Cary, NC). All *p* values are two-sided.

## Results

### Population characteristics of case-cohort study

The majority were negative for HBeAg (90.7%) and genotype B HBV (78.4%) infected. There were no significant differences in any of HBV-related factors between HBV-monoinfected and HBV/HCV dual-infected men. The mean and median follow-up time was 8.1 (SD = 4.0) and 8.3 (range: 0.6–18.8) years, respectively, for men who progressed to HCC, and 19.1 (SD = 2.3) and 19.3 (range: 2.1–21.3) years, respectively, for noncases. Compared with noncase subjects, men who progressed to HCC were older and had higher viral load at recruitment (*p*<0.0001). Case subjects were also more frequently smokers, BMI≥25 kg/m^2^, HBeAg positive, genotype C HBV infected, and ALT elevated (*p*≤0.01), as well as had higher proportions of a first-degree family history of HCC and liver cirrhosis detected by ultrasonography during follow-up (*p*<0.0001). Distribution of baseline plasma sPD-1 was similar between HBV-monoinfected and HBV/HCV dual-infected men (*p* = 0.2482). The HCC cases had higher plasma sPD-1 compared with noncase subjects (*p* = 0.0014) (Table 1).

**Table 1:** Baseline characteristics of study participants in the case-cohort study according to infection status.

Characteristics	HBV Monoinfection			Dual HBV/HCV Infection	Mono- vs Dual infection
	All (n = 1137)	HCC (n = 123)	Noncases (n = 1014)	All (n = 144)^a^	*p* value
**Age at entry, years, median (IQR)**	43.2 (38.2–51.3)	50.7^b^ (43.4–60.8)	42.5 (37.8–49.9)	43.3 (39.0–51.9)	0.6990
**ALT>ULN, %**	8.6	27.1^b^	6.3	9.8	0.6361
**HBeAg positivity, %**	9.8	16.5^b^	9.0	5.6	0.1260
**Median HBV DNA levels, log copies/mL (IQR)**	4.02 (3.04–5.57)	6.22^b^ (4.25–7.76)	3.91 (2.95–5.23)	4.03 (3.05–5.34)	0.2290
**HBV genotype, %**					0.1398^c^
C	18.0	50.8^b^^,^ c	13.9	12.5	
B	77.6	44.3	81.7	85.2	
B+C mixed type/A	4.4	4.9	4.4	2.3	
**First-degree family history of HCC, %**	7.6	17.9^b^	6.3	4.9	0.3060
**Cigarette smoking, %**	31.3	42.3^b^	30.0	25.7	0.1802
**Alcohol consumption, %**	21.0	25.2	20.5	18.1	0.4461
**BMI≥25 kg/m^2^, %**	30.7	40.7^b^	29.5	25.7	0.2475
**Plasma sPD-1 levels, pg/mL, median (IQR)**	293.3 (126.0–663.6)	394.8^b^ (185.6–906.9)	282.0 (117.3–637.6)	317.2 (106.8–936.4)	0.2482

Four HBV-monoinfected subjects (1 case and 3 noncases) and 1 HBV/HCV dual-infected subject had missing data on ALT levels; 27 HBV-monoinfected subjects (2 cases and 25 noncases) had missing data on HBeAg status; 17 (1 case and 16 noncases) HBV monoinfected and 16 (1 case and 15 noncases) HBV/HCV dual infected subjects had missing data on HBV genotype.

a Containing 3 HCC cases diagnosed during follow-up.

b *P*<0.05 for comparison between HCC cases and noncases.

c *P* value for testing the difference in the proportion of genotype C HBV infection between groups.

IQR, interquartile range; ULN, upper limit of normal.

### sPD-1 and HCC

For Asians, it has been reported that HBV/HCV dual-infected patients mainly presented HBV-dominant dual infection [24]. In this study, HBV-monoinfected and HBV/HCV dual-infected participants also revealed similar viral features of HBV and sPD-1 levels. To gain statistical power, we thereafter combined the two groups in the following analyses performed to assess the relationship of sPD-1 with HCC.

The median age at diagnosis of HCC was 59.8 years (range = 39.8–81.3 years) for cohort-based case subjects and 51.0 years (range = 20–73 years) for hospital-based case subjects. In the case-cohort study, the ORs of HCC for increasing sPD-1 quartiles were 1.0 (referent), 1.83 (95% CI = 0.95–3.52), 2.64 (95% CI = 1.43–4.91), and 2.81 (95% CI = 1.52–5.17), respectively, after adjustment for age, cigarette smoking, and alcohol consumption. Regardless of various additional variables that were controlled for, sPD-1 was still significantly associated with HCC.

A similar result was observed for male (vs. Q1: ORs were 1.25 [95% CI = 0.87–1.80] in Q2, 1.53 [95% CI = 1.09–2.16] in Q3, and 2.37 [95% CI = 1.70–3.30] in Q4, respectively) but not female case patients with existing HBV-related HCC in the case-control analysis, in which male cases had significantly higher median levels of sPD-1 than female cases (443.3 vs. 307.3 pg/mL) (*p* = 0.0074). Higher sPD-1 appeared to be associated with an increased risk of HCC for male cases regardless of clinical stage, α-fetoprotein or age-of-onset, and there seems to be presence of a threshold effect at 282 pg/mL of sPD-1 (Table 2).

**Table 2:** Risk of hepatocellular carcinoma (HCC) by quartile (Q) of plasma sPD-1 levels: case-cohort and case-control studies^a^.

	Plasma sPD-1 (pg/mL)								
	Q1 (≤117.3)		Q2 (117.4–282.0)		Q3 (282.1–637.6)		Q4 (>637.6)		*p* for trend
	No.	OR	No.	OR (95% CI)	No.	OR (95% CI)	No.	OR (95% CI)	
**Non-HCC participants**	291		275		290		299		
**Cohort-based HCC case subjects**	16		27		39		44		
Model 1		1.00		1.83 (0.95–3.52)		2.64 (1.43–4.91)		2.81 (1.52–5.17)	0.0004
Model 2^b^		1.00		1.60 (0.81–3.14)		2.26 (1.19–4.29)		2.42 (1.28–4.56)	0.0035
Model 3^c^		1.00		1.51 (0.75–3.03)		2.15 (1.12–4.13)		2.29 (1.20–4.38)	0.0070
**Hospital-based HCC case subjects** d	114	1.00	121	1.09 (0.79–1.50)	161	1.34 (0.99–1.82)	218	1.81 (1.35–2.44)	<0.0001
Female	31	1.00	22	0.64 (0.35 –1.17)	31	0.88 (0.51–1.53)	30	0.71 (0.41–1.24)	0.4140
Male	83	1.00	99	1.25 (0.87–1.80)	130	1.53 (1.09–2.16)	188	2.37 (1.70–3.30)	<0.0001
**Clinical stage**									
Early-stage HCC^e^	39	1.00	50	1.36 (0.85–2.18)	64	1.62 (1.03–2.55)	98	2.47 (1.61–3.79)	<0.0001
Late-stage HCC	44	1.00	49	1.19 (0.74–1.89)	66	1.50 (0.96 –2.33)	90	2.36 (1.54–3.62)	<0.0001
**Serum α-fetoprotein**									
<400 ng/mL	46	1.00	63	1.42 (0.91–2.21)	76	1.60 (1.04–2.45)	119	2.56 (1.70–3.86)	<0.0001
≥400 ng/mL	37	1.00	36	1.06 (0.64–1.76)	54	1.53 (0.96–2.44)	69	2.17 (1.38–3.42)	0.0002
**HCC onset age, years**									
<50	43	1.00	46	1.21 (0.75–1.93)	56	1.37 (0.87–2.15)	76	2.20 (1.43–3.39)	0.0003
≥50	40	1.00	53	1.27 (0.74 –2.18)	74	1.89 (1.13–3.14)	112	2.48 (1.53–4.05)	<0.0001

a ORs and 95% CIs were mutually adjusted for age (continuous variable), cigarette smoking (yes or no), and alcohol consumption (yes or no), except for models 2 & 3.

b Model 2: ORs and 95% CIs were mutually adjusted for age (continuous variable), cigarette smoking (yes or no), alcohol consumption (yes or no), first-degree family history of HCC (yes or no), BMI (≥25 or <25 kg/m^2^), and HBV viral load (≥4.39 or <4.39 log copies/mL).

c Model 3: ORs and 95% CIs were mutually adjusted for age (continuous variable), cigarette smoking (yes or no), alcohol consumption (yes or no), first-degree family history of HCC (yes or no), BMI (≥25 or <25 kg/m^2^), HBV viral load (≥4.39 or <4.39 log copies/mL), and ALT>ULN (yes or no).

d ORs and 95% CIs for HCC according to sPD-1 quartile levels were derived from comparisons between the entire or subgroups (as indicated below) of hospital-based cases vs. the 1155 noncases from the case-cohort study.

e Defined as solitary tumor ≤5 cm or <4 lesions and none>3 cm.

CI, confidence interval; OR, odds ratio; ULN, upper limit of normal.

### sPD-1 and dynamics of viral load

**Figure 2:**
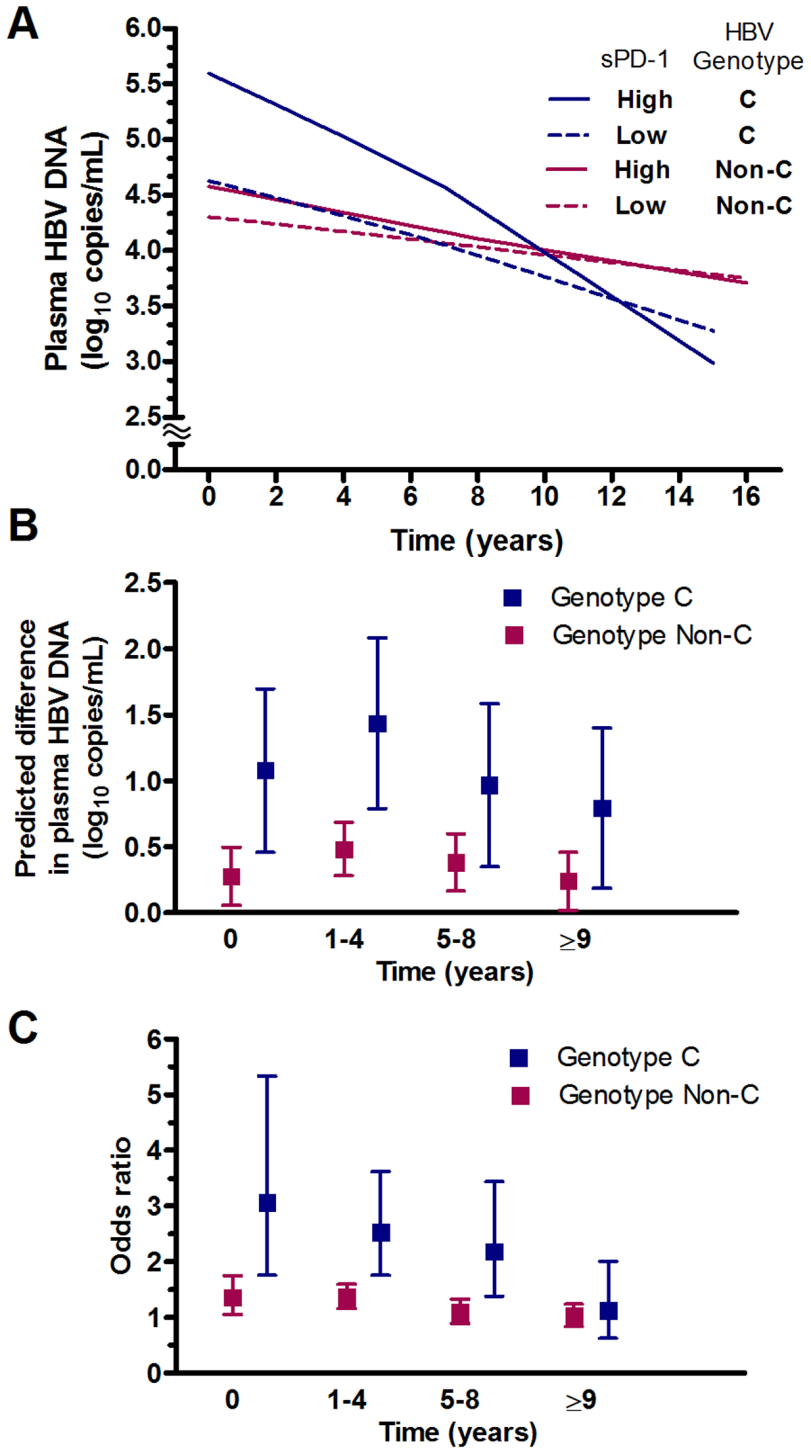
Effect of plasma sPD-1 on dynamics of HBV viral load across years of follow-up. (A) Plots of predicted mean plasma HBV-DNA levels derived from LOESS for each year of follow-up time according to different combinations of sPD-1 levels and HBV genotype. (B) Predicted difference (regression coefficient; squares) with 95% confidence interval (bars) in plasma HBV-DNA levels between high (>282 pg/mL) and low sPD-1 groups across time by HBV genotype. (C) Odds ratios (squares) and 95% confidence intervals (bars) of having a high viral load (≥4.39 log copies/mL) for high vs. low sPD-1 across time by HBV genotype.

Since PD-1 expression is associated with poor control of viral replication [8]–[12], we next evaluated the association between baseline sPD-1 levels and longitudinal viral load in the case-cohort study. LOESS model revealed that longitudinal viral load varied according to sPD-1 levels and HBV genotype (Figure 2A). High sPD-1 levels (>282 pg/mL) were positively associated with longitudinal viral load (*p*<0.04) over time (Figure 2B) and maintenance of a higher viral load of ≥4.39 log copies/mL within 4 years (*p*<0.02) (Figure 2C) for each class of HBV genotype. The effect of sPD-1 on viral load appeared to be greater and associated with longer duration of persistence of higher viral load (OR for persistence remained significant for up to 8 years after baseline in genotype C but not in genotype non-C group) in HBV genotype C vs. non-C genotype group (Figure 2C).

### Interaction of sPD-1, viral load, and HBV genotype

There was significant additive interaction (indicated by RERI, AP, and synergy index) between high sPD-1 and high viral load at baseline in regard to HCC (OR = 6.29, 95% CI =  3.48–11.37) or liver cirrhosis (OR = 7.87, 95% CI = 4.27–14.50) risk. The joint effect of these two factors was greater when combined with genotype C HBV infection (OR for HCC = 30.47, 95% CI = 16.35–56.79; OR for liver cirrhosis = 18.87, 95% CI = 10.36–34.35), with a RERI as high as 27.08 (95% CI = 8.76–45.41) derived from the relation with HCC (Table 3).

**Table 3:** Interactions of high sPD-1 with high HBV viral load and copresence of these two risk factors with genotype C HBV in risks for HCC and liver cirrhosis.

Group		HCC	Non-HCC				LC	Non-LC			
		No.	No.	OR^a^	(95% CI)	*p* value	No.	No.	OR^a^	(95% CI)	*p* value
**HBV viral load**	**sPD-1**										
Low	Low	16	371	1.00			14	349	1.00^b^		
Low	High	18	342	1.29	(0.64–2.63)	0.4784	18	324	1.38	(0.67–2.85)	0.3830
High	Low	27	195	2.96	(1.53–5.76)	0.0013	29	176	3.87	(1.97–7.59)	<0.0001
High	High	65	247	6.29	(3.48–11.37)	<0.0001	69	218	7.87	(4.27–14.50)	<0.0001
*RERI (95% CI)*		3.03 (0.48–5.58)					3.62 (0.48–6.76)				
*AP (95% CI)*		0.48 (0.20–0.76)					0.46 (0.19–0.73)				
*Synergy index (95% CI)*		2.34 (1.06–5.20)					2.11 (1.09–4.11)				
**Copresence of high sPD-1 and high HBV viral load**	**HBV genotype**										
No	Non-C	39	756	1.00^c^			42	711	1.00^d^		
Yes	Non-C	23	213	1.94	(1.11–3.39)	0.0206	32	184	2.85	(1.73–4.70)	<0.0001
No	C	20	123	2.45	(1.34–4.48)	0.0035	19	111	2.40	(1.32–4.35)	0.0040
Yes	C	42	32	30.47	(16.35–56.79)	<0.0001	36	33	18.87	(10.36–34.35)	<0.0001
*RERI (95% CI)*		27.08 (8.76–45.41)					14.62 (3.96–25.28)				
*AP (95% CI)*		0.89 (0.81–0.97)					0.78 (0.63–0.92)				
*Synergy index (95% CI)*		12.33 (5.09–29.88)					5.50 (2.57–11.75)				

a ORs and 95% CIs were mutually adjusted for age (continuous variable), cigarette smoking (yes or no), alcohol consumption (yes or no), first-degree family history of HCC (yes or no), and BMI (≥25 or <25 kg/m^2^).

b 84 subjects with missing data on ultrasonography measurement of liver during follow-up were excluded from analysis.

c 33 subjects (2 HCC cases and 31 non-HCC subjects) with missing data on HBV genotype were excluded from analysis.

d 113 subjects were excluded due to missing data on follow-up ultrasonography measurement of liver and/or HBV genotype (80 were missing on follow-up ultrasonography measurement alone; 29 were missing on HBV genotype alone; 4 were missing on both variables).

AP, attributable proportion due to interaction; CI, confidence interval; HCC, hepatocellular carcinoma; LC, liver cirrhosis; OR, odds ratios; RERI, relative excess risk due to interaction.

## Discussion

We found that high sPD-1 levels (defined as >282 pg/mL) determined at baseline was associated with a 2-fold increase in the risk of developing HCC during a median follow-up of 19.3 years. A similar result was obtained for male patients with HBV-related HCC recruited after diagnosis on a comprehensive basis, and the relationship did not vary substantially according to age-of-onset and clinical prognostic factors. Sustained PD-1 expression is associated with impaired T-cell immunity in not only HBV but also HCV infection [25]. Our result showing that HBV-monoinfected and HBV/HCV dual-infected participants shared similar distribution of sPD-1 is thus biologically plausible. Monitoring viral load in conjunction with ALT has been proven to provide prognostic significant information in hepatitis B [26]. We found that levels of sPD-1 were associated HCC risk even after adjustment for viral load and ALT. We indeed also detected an interactive additive effect of elevated sPD-1 and high viral load on subsequent risks for HCC and liver cirrhosis. Moreover, combining both conditions with HBV genotype C infection greatly increased the risk of HCC.

Our finding of a positive association of sPD-1 and HBV viral load was consistent in the same direction for HCC risk. This finding is important because viral load has been firmly established as an important determinant for HBV-related HCC [2]–[4], and is also biologically plausible because PD-1 expression level on virus-specific CD8+ T cells was positively associated with plasma viral load in patients with chronic hepatitis B [12]. Our previous studies, from a longitudinal analysis of repeated measures of viral load over 16 years, demonstrated that prolonged maintenance of high levels of viral load is more important than transient increases of viral load in predicting risk of HCC [3], [27]. Using the longitudinal viral-load data, the present study has further shown that high sPD-1 (defined as >282 pg/mL) predicted higher viral load for 4 or more years regardless of HBV genotype. Thus, the baseline determination of sPD-1 is predictive of persistently high viral load and an increased risk of future HCC event even after long-term follow-up.

HBV genotype C has been associated with poor clinical outcomes, including HCC, and sustained high viral load, compared to genotype B [2]–[4], [27]. The mechanisms underlying the pathogenic difference between HBV genotypes remain unclear but may involve effect of genotype-specific sequence variation in virus-host immunity interplay. There is significant sequence variation in the T-cell epitopes between HBV genotypes [28]. Evidence has suggested that virus epitope-specific T-cell responses correlate with PD-1 expression, and epitope escape mutations can affect the process of T-cell exhaustion in response to up-regulated PD-1 [8], [29].

Along these lines, we have also examined whether there is differential effects of sPD-1 on viral load between HBV genotypes. sPD-1>282 pg/mL was observed to be associated with greater increase of viral load and longer duration of maintenance of a higher viral load, which has been associated with elevated HCC risk [2], [3], in HBV genotype C- compared to non-C (mostly genotype B)-infected men. Furthermore, there is evidence for an additive interaction between genotype C and high levels of both sPD-1 and viral load in relation to disease progression. Currently, there are only a handful of laboratory variables (eg, ALT, HBeAg, viral load, and HBsAg levels) that may carry information for predicting long-term risk of HCC in chronic HBV infection. Given the magnitude of the relative risk estimate for this interactive effect (Table 3), testing sPD-1 levels among those with genotype C HBV would be useful not only to enable cost-effectiveness of tumor surveillance but also to prioritize patients for antiviral therapy.

HCC occurs mainly in men [30]. Multiple mechanisms have been proposed for this gender disparity, including gender difference in inflammatory cytokine production [31]. In this study, we also observed striking gender difference in the sPD-1 levels and with higher levels in male than in female HCC patients. However, we did not include unaffected women. On applying plasma sPD-1 to clinical practice, further research is needed to determine whether gender difference exists in the distribution of sPD-1 levels in population-based sample and whether sPD-1 risk thresholds should be adjusted for different gender groups.

Strengths of this study included its using stored blood samples collected in a prospective study and large size, which is critical to determine whether a biomarker can serve as predictive markers for the risk of developing a disease. It is known that the case-cohort study is a variation of the nested case-control study. To evaluate the efficiency for this study, we calculate the asymptotic relative efficiency defined previously according to the theory of the case-control study design [32], and the value is 0.9. Furthermore, this study contains an additional case series, which provides further evidence to justify the use of plasma sPD-1 as a marker of immunity for chronic hepatitis B. Because the availability of detailed information on viral and clinical features, including viral load updated at multiple time points, we could evaluate and exclude important confounding variables, as well as comprehensively examine the effect of sPD-1 on the key steps towards HCC.

A potential limitation of this work is that the source and specific function of sPD-1 are not known. In contrast to our findings, utilization of recombinant mouse sPD-1 with other molecule has been shown to block the PD-1-PD-L interaction and enhance antitumor immunity in an animal model for HCC [18]. However, there have been many instances that animal models do not reliably predict human outcomes because of the biological differences between species [33]. The relationship between PD-1 expression on T cells and plasma sPD-1 levels in people with chronic HBV infection deserve further investigation. Our finding that the baseline measurement of plasma sPD-1 carries significant long-term prognostic information in hepatitis B is intriguing, but the dynamics of sPD-1 is necessary to be addressed before it is incorporated into clinical practice.

In conclusion, this study demonstrated that the significance of a single, random sPD-1 assay in predicting HCC risk in people with chronic HBV infection. The increased levels of sPD-1 seen in plasma from chronic hepatitis B patients are of clinical relevance as the PD-1 levels correlated with viral load and disease progression in people with chronic viral infection [8]–[12], [25], [29]. Further studies are needed to elucidate the precise mechanisms underlying the observed association between sPD-1 and HCC.
